# Did you know? State-of-the-art preprocessing diffusion MRI data can improve tractography

**DOI:** 10.1007/s00429-026-03107-7

**Published:** 2026-03-30

**Authors:** Kurt G. Schilling, Matthew Cieslak, Maxime Descoteaux, Bennett A. Landman, Franco Pestilli, Ariel Rokem, Stamatios N. Sotiropoulos, Jacques-Donald Tournier, Jelle Veraart

**Affiliations:** 1 Vanderbilt University Medical Center, Nashville, TN USA; 2 Vanderbilt University Institute of Imaging Science, Nashville, TN USA; 3 Lifespan Informatics and Neuroimaging Center (PennLINC), Department of Psychiatry, Perelman School of Medicine, University of Pennsylvania, Philadelphia, PA USA; 4 Penn/CHOP Lifespan Brain Institute, Perelman School of Medicine, Children’s Hospital of Philadelphia Research Institute, Philadelphia, PA USA; 5 Department of Psychiatry, Perelman School of Medicine, University of Pennsylvania, Philadelphia, PA USA; 6 Sherbrooke Connectivity Imaging Lab (SCIL), Department of Computer Science, Universite de Sherbrooke, Sherbrooke, QC Canada; 7 Department of Electrical and Computer Engineering, Vanderbilt University, Nashville, TN USA; 8 Department of Computer Science, Vanderbilt University, Nashville, TN USA; 9 Department of Psychology, Department of Neuroscience, Center for Learning and Memory, Center for Perceptual Systems, The University of Texas, Austin, TX USA; 10 Department of Psychology, University of Washington, Seattle, WA USA; 11 The University of Washington eScience Institute, University of Washington, Seattle, WA USA; 12 Department of Biomedical Engineering, School of Biomedical Engineering and Imaging Sciences, King’s College London, King’s Health Partners, St. Thomas’ Hospital, London, UK; 13 Centre for the Developing Brain, School of Biomedical Engineering and Imaging Sciences, King’s College London, King’s Health Partners, St. Thomas’ Hospital, London, UK; 14 Sir Peter Mansfield Imaging Centre, Mental Health and Clinical Neurosciences, School of Medicine, University of Nottingham, Nottingham, UK; 15 Nottingham Biomedical Research Centre, Nottingham University Hospitals NHS Trust, Queen’s Medical Centre, Nottingham, UK; 16 Center for Biomedical Imaging, Department of Radiology, NYU Grossman School of Medicine, New York, NY USA; 17 Center for Advanced Imaging Innovation and Research (CAI2R), Department of Radiology, New York University Grossman School of Medicine, New York, NY USA

**Keywords:** Tractography, Preprocessing, Fiber orientation distribution, Denoising, Artifacts, Distortion correction

## Abstract

Diffusion MRI fiber tractography is sensitive to noise and artifacts in diffusion-weighted images, and these challenges can propagate into fiber-orientation estimation and the tractography process. In this “Did You Know” communication, we synthesize evidence that state-of-the-art preprocessing improves tractography anatomical fidelity and test-retest reproducibility compared to minimally processed data. We summarize best-practice preprocessing – including denoising, motion and eddy current correction, EPI distortion correction, and Gibbs ringing removal – along with additional and emerging steps, and highlight integrated, publicly available pipelines that implement these methods in standardized, containerized workflows. We also outline practical acquisition and data-handling considerations that maximize the benefits of modern processing, providing a foundation for reliable tractography-based studies of the brain.

## Introduction

**Fig. 1:**
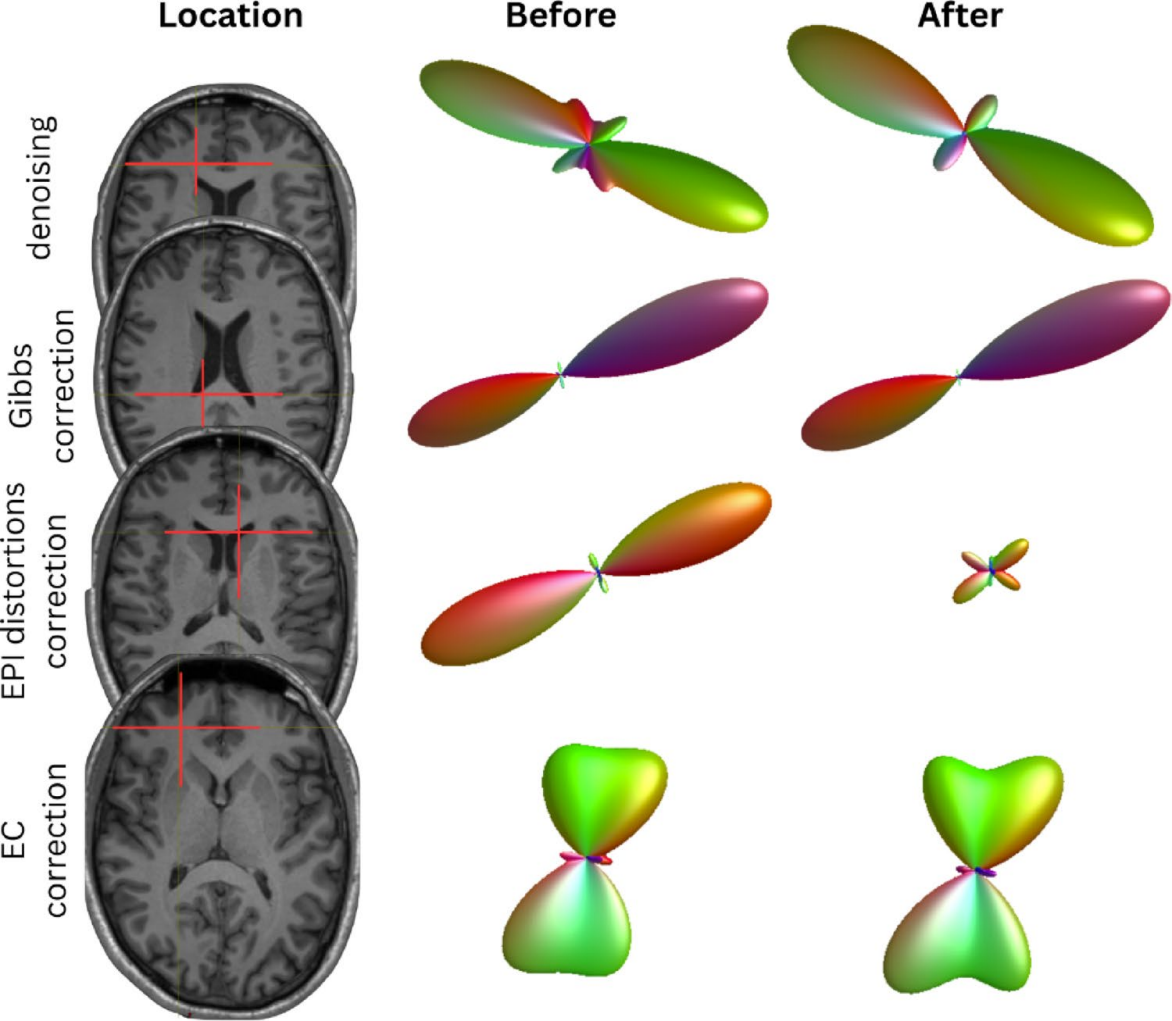
We show the impact of individual preprocessing steps (denoising, Gibbs ringing correction, susceptibility-induced geometric or “EPI” distortion correction, and eddy-current (EC) distortion correction on fiber orientation distribution functions (fODFs). For each preprocessing step (rows), an example voxel location is indicated on an anatomical image (cross-hairs), and the corresponding fODF is shown before (left) and after (right) applying the indicated correction. Denoising has been shown to reduce the number of spurious fibers (Veraart et al. 2016a, b), Gibbs ringing correction results in sharper signal kernels and fODFs (Kellner et al. 2016), susceptibility-induced geometric distortion correction improves alignment of diffusion and structural MRI data (Andersson et al. 2003), lowering the risk of streamline traversing the ventricles for example, and eddy current distortions sharpen the fODFs by reducing signal scattering across dMRI data

Diffusion MRI (dMRI) tractography offers unprecedented insights into brain connectivity (Behrens and Sporns 2012; Glasser et al. 2016; Jbabdi et al. 2015; Maier-Hein et al. 2017), but its reliability depends critically on preprocessing of the diffusion-weighted MR images prior to further analyses (Tax et al. 2022; Tournier et al. 2011). The analysis of dMRI data is subject to various sources of variability that relate to the imaging hardware, imaging protocol, or analysis software (Schilling et al. 2021; Warrington et al. 2025). Such variability might impact the reproducibility of findings or challenge the harmonization of data for pooled analyses across sites or studies (Cetin-Karayumak et al. 2024). However, each individual dMRI data set intrinsically suffers from noise and artifacts that distort the geometry and signal of the images. Quality control or optimized acquisition strategies are recommended, but insufficient to mitigate the impact of imaging artifacts on tractography. Correcting motion and imaging artifacts and reducing thermal noise are critical computational steps, collectively *referred to as preprocessing*, to prepare data for further analysis. Fortunately, building upon the FAIR principles (Findable, Accessible, Interoperable, and Reusable; (Wilkinson et al. 2016) and data standards, a new generation of robust, publicly available preprocessing pipelines make state-of-the-art corrections readily accessible (Cieslak et al. 2021; Chen et al. 2024; Dhiman et al. 2024; Theaud et al. 2020; Hsu et al. 2023; Cruces et al. 2022; Glasser et al. 2013; Irfanoglu et al. 2025; Cai et al. 2021). These pipelines integrate a series of widely-adopted steps, including but not limited to denoising, Gibbs ringing removal, motion/eddy current correction with *b*-vector rotation and EPI distortion correction (Fig. 1) in open-source software workflows, oftentimes containerized, to maximize reproducibility and interoperability, while lowering barriers to entry.

We synthesize the literature on each key preprocessing component and its value for tractography, and highlight how these pipelines already improve tractography outcomes over unprocessed data. Even as development and validation efforts continue, applying these methods yields considerably more anatomically accurate and reliable fiber reconstructions than leaving data uncorrected. We advocate that neuroimaging researchers adopt such pipelines and keep investing in their further development to ensure higher-quality tractography, recognizing that while challenges remain, the current practices represent a major leap forward for brain mapping.

## Best-practice preprocessing steps and their benefits for tractography

### Denoising

Noise in DWIs degrades signal-to-noise ratio (SNR) and can propagate into downstream diffusion metrics and tractography. Denoising has been shown to translate to more precise diffusion metrics and tractography – improving test–retest reproducibility of diffusion-derived measures (Manzano Patron et al. 2024; Veraart et al. 2016a, b), while also advancing the performance of motion correction algorithms (Cieslak et al. 2024). In the context of tractography, denoising facilitates more accurate and reproducible fiber orientation estimates (Maier-Hein et al. 2017) with fewer spurious or missed fiber directions (Veraart et al. 2016a, b) (Fig. 1), which intuitively leads to more accurate and reproducible streamline generation. Because of this, pipelines like QSIPrep (Cieslak et al. 2021), TractoFlow (Theaud et al. 2020), PreQual (Cai et al. 2021), Designer (Chen et al. 2024), IdIO (Hsu et al. 2023), Tortoise (Irfanoglu et al. 2025), and PyDesigner (Dhiman et al. 2024) perform denoising as the first step on raw DWIs. Today, Marchenko–Pastur principal component analysis (MP-PCA) denoising (Veraart et al. 2016a, b) and Patch2Self (Fadnavis, Batson, and Garyfallidis 2020) have emerged as commonly-used nonparametric methods to suppress random noise. These tools are already publicly available through their implementation in software libraries such as MRtrix (J.-D. Tournier et al. 2019) and DIPY (Garyfallidis et al. 2014) and various pipelines. Yet, there are ongoing efforts to further improve their efficacy, for instance by denoising in the complex domain and as part of the image reconstruction (Manzano Patron et al. 2024; Moeller et al. 2021; Cordero-Grande et al. 2019). The latter not only addresses noise variance reduction, but can have a significant effect in reducing biases from the noise floor that affects all dMRI-derived measures, including fiber orientations (Jones and Basser 2004; Eichner et al. 2015).

### Motion and Eddy current correction

Head motion and eddy current–induced distortions are major sources of error in diffusion MRI (Anderson and Gore 1994), leading to misalignment of volumes and subsequent modeling and tractography inaccuracies (Yendiki et al. 2014) (Fig. 1). A first approach to mitigate these issues is to use pairwise image registrations between the DWI volumes, typically modelling both motion and eddy current distortion fields as linear affine transformations (Rohde et al. 2004; Jezzard et al. 1998). Importantly, diffusion gradient vectors should be rotated accordingly during motion correction (Alexander Leemans and Jones 2009), or else the orientation data would no longer correspond to the true diffusion sensitization direction after the image has been rotated/aligned, and can lead to both false positive and false negative tractography results (Maier-Hein et al. 2017).

Modern pipelines extend these ideas and estimate motion parameters concurrently with eddy current distortion fields considering all data at once (Andersson and Sotiropoulos 2016). This allows subsequent alignment of the DWI volumes by inverting these fields, as well as higher order models for the distortion fields to be considered (quadratic, cubic). They further enable identification of outliers, such as signal dropouts due to motion (Andersson et al. 2016). State-of-the-art tools, such as FSL’s Eddy (Andersson and Sotiropoulos 2016), are aimed for *shelled* data (i.e., diffusion directions sampled on one or more spherical shells at fixed b-values) and are integrated into publicly available pipelines. Alternative approaches, including mutual information-based registration techniques that are regularized by eddy-current specific transformation models or iteratively created target images based on model predictions (Cieslak et al. 2021; Legarreta Gorroño et al. 2025) are available for data that has not been acquired with isotropically distributed gradient directions on one or more *b*-shells.

It is important to note that in the presence of heavy motion dealing only with between-volume alignment may not be adequate (e.g. in the case of neonatal tractography (Bastiani 2019). In such cases, within-volume slice-wise motion correction must be considered and performed (Andersson et al. 2017; Christiaens et al. 2021). Doing so generally requires slice acquisition timing/order information, which may not be easily inferred for multiband/interleaved protocols and should be carried forward explicitly via the sequence metadata and BIDS sidecars.

### EPI distortion correction for anatomical fidelity

Echo-planar imaging (EPI) distortions, caused by magnetic susceptibility differences, can stretch or compress diffusion-weighted images (DWIs), leading to misalignment with anatomical MRI. They also create challenges in correcting motion and eddy currents as all three interact (Andersson et al. 2018). These image deformations manifest most obviously as signal intensity pile-ups, though other parts of the brain will typically also be affected. Together, these distortions can cause tractography streamlines to deviate from their true paths, and challenge the alignment of gray matter white matter interface relative to the tractogram, resulting in inaccurate connectivity inferences (Fig. 1). It has been convincingly shown that not correcting for distortions can produce significant (inaccurate) deviations in major pathways (Irfanoglu et al. 2012; Embleton et al. 2010; Yang et al. 2022), and that applying distortion correction improves tract consistency and accuracy, with a conclusion worth repeating “add an EPI distortion correction step to the diffusion MRI processing pipeline if the output is to be used for fiber tractography.” (Irfanoglu et al. 2012). To mitigate these effects, best-practice pipelines apply susceptibility distortion correction (SDC). A common recommendation is to acquire, minimally, a pair of reverse b = 0 images (e.g. one Anterior-to-Posterior and one Posterior-to-Anterior) to serve as an alternative to an off-resonance field map. Tools such as tools such as FSL’s topup (Andersson, Skare, and Ashburner 2003) or Tortoise’s DR-BUDDI (Irfanoglu et al. 2015) can estimate the off-resonance field from this set of images distorted in equal, yet opposite directions. However, it is important to note that the amount of reverse-phase encoded data influences correction quality. Recent literature suggests a hierarchy of efficacy: while a single b = 0 pair is sufficient for geometric correction, acquiring a full diffusion dataset (i.e., both b = 0 and DWIs) with opposing phase-encoding directions allows more robust signal restoration (Irfanoglu et al. 2021). At the highest level, examples of four-way phase encoding (AP, PA, LR, and RL) have been shown to yield the highest reproducibility and anatomical fidelity, mitigating both distortion and additional artifacts (ghosting) by leveraging data from unaffected directions (Thai et al. 2025; Irfanoglu et al. 2021). While definitive comparisons of distortion corrections are missing, especially in the context of tractography, evidence suggests that phase-encoding methods generally outperform field map-based methods (Gu and Eklund 2019; Cercignani et al. 2007). Finally, if no fieldmap or reverse phase encoded data is present, alternatives like Synb0-DisCo (synthetic distortion correction via deep learning) (Kurt G. Schilling et al. 2019) can be applied. Distortion correction facilitates better alignment to structural images and anatomical boundaries, improves normalization with standard templates, reduces tract reconstruction errors, increases reproducibility of pathway identification across scans, and is critical for anatomical fidelity in tractography.

### Gibbs ringing removal

Finite *k*-space sampling results in oscillatory intensity ripples around *edges* in the DW data. This artifact, commonly referred to as Gibbs ringing, can distort diffusion orientations and lead to inaccurate fiber orientation estimates (Veraart et al. 2016a, b) (Fig. 1). Correcting these artifacts before modeling fiber orientation distributions is considered best practice. Gibbs correction improves diffusion metrics by eliminating oscillatory intensity bias (Kellner et al. 2016), reducing spurious high-frequency structures that may be misinterpreted as multiple fiber orientations, particularly near tissue interfaces and CSF boundaries. This correction enhances the precision of diffusion tensor and fiber orientation distribution estimations, contributing to more reliable tractography results (Mazur-Rosmus and Krzyżak 2024). While initial approaches were based on the regularized minimization of total variation, more recently, the sub-voxel shift method developed by Kellner et al. (2016) (Kellner et al. 2016) suppresses ringing in a computationally efficient way, while lowering the dependency on user-defined settings. The method is widely accessible as it is implemented in software libraries and pipelines, see above. However, in its original design, the application of the method is technically limited to data acquired without partial fourier. More general solutions have recently been developed, but further efforts for its evaluation and dissemination are needed (Lee, Novikov, and Fieremans 2021).

### Other preprocessing steps

While denoising, Gibbs correction, motion and distortion corrections are commonly adopted in accessible pipelines, there are various additional steps that can be considered to further clean the data prior to analyses. These steps include, but are not limited to B1 inhomogeneity correction, signal drift correction, Rician bias correction, gradient nonlinearity correction, and spatial resolution upsampling (Tax et al. 2022). Each of these steps holds the potential to improve the accuracy and precision of tractography and connectivity analyses.

- *B1 inhomogeneity*: Smooth, non-anatomical signal intensity variations in MRI images are often attributed to B1 inhomogeneity, which arises from imperfections in RF coil uniformity and intrinsic properties of high-field imaging. This artifact gains less attention in dMRI compared to other modalities because it is often mitigated by normalizing diffusion-weighted images with the corresponding non-diffusion-weighted image, assuming minimal motion. However, when normalization using *b* = 0 images is not preferred or if strong subject motion is present, explicit correction strategies may be required. B1 bias fields are typically estimated as smooth multiplicative fields using automated techniques like histogram-based optimization (Tustison et al. 2010; Sled and Pike 2000) or adaptive low-pass filtering (Zhang, Brady, and Smith 2001). Software packages such as MRtrix, FSL offer B1 bias field correction, and some pipelines, e.g. Designer and QSIprep, do provide B1 inhomogeneity as an optional step. However, the procedure introduces scaling-based heteroscedasticity that should be carefully addressed during subsequent analyses to ensure robust parameter estimation.
- *Signal drift correction*: During long dMRI acquisition, there is a risk for a global and monotonic (gradual) signal change over time due to temperature-induced variations in the magnetic field strength (Vos et al. 2017; Hansen et al. 2019). Such gradual signal fluctuations will impact the quantification of dMRI features in protocol-dependent ways. While there are increasing efforts to mitigate these challenges through dynamic field corrections during data acquisition, it is recommended to assess signal stability by plotting the mean signal of interspersed non-diffusion-weighted (b = 0) images (e.g., within a brain mask) as a function of acquisition time/volume index. Drift can be quantified by fitting a simple linear (or quadratic) trend to these b = 0 means and using the fitted trend to derive a single multiplicative scaling factor for each volume; applying this per-volume “global rescaling” stabilizes the signal across the series *(*Vos et al. 2017).
- *Rician bias correction*: Magnitude MRI data is prone to SNR-dependent signal biases, often referred to as *Rician biases*. At lower SNR, the signal tends to appear higher than its true underlying value (Gudbjartsson and Patz 1995). In dMRI, SNR is b-value and directionally dependent. When not accounted for, these Rician biases tend to lower diffusivities, fractional anisotropy, or orientation distribution functions (Jones and Basser 2004). While various solution strategies have been proposed, they are not routinely adopted in data preprocessing pipelines, in part because correcting signal biases depend on prior knowledge of noise level (Koay, Özarslan, and Basser 2009), exact data distribution that depends on coil configurations and reconstruction algorithms (Dietrich et al. 2008), and preceding preprocessing steps (Veraart et al. 2013).
- *Gradient nonlinearity correction*: Gradient coils in MRI systems often exhibit spatially varying nonlinearities, particularly away from the magnet isocenter, which can cause geometric distortions and bias diffusion encoding strength (Rudrapatna et al. 2021). These deviations can impact dMRI analyses, causing errors in diffusion tensor metrics, orientation distribution functions, and tractography. Gradient nonlinearities are typically mapped using spherical harmonic expansions or phantom imaging, often performed by scanner manufacturers or in-house teams. Corrections are applied either prospectively during image reconstruction or retrospectively in preprocessing pipelines, using methods that estimate effective gradient strengths, apply distortion correction, and adjust *b*-matrices accordingly. High-gradient systems (Ramos-Llordén et al. 2025; Foo et al. 2020) and significant head motion exacerbate these effects, necessitating careful correction. Today, few modern pipelines address gradient nonlinearities either jointly or sequentially with other distortions, in part because of the dependency on scanner-specific information and computational expense of regional b-matrix adjustments.
- *Spatial resolution upsampling*: Highly anisotropic voxels can introduce systematic biases that underestimate anisotropy in crossing fibers and hinder the reconstruction of branching/curving structures in tractography (Neher et al. 2013; Oouchi et al. 2007). To mitigate this, resampling to high-resolution isotropic grid (Dyrby et al. 2014) can be performed as a final preprocessing step (Theaud et al. 2020) to improve delineation of anatomical boundaries and reproducibility of reconstructed bundles (McMaster et al. 2025).

While any of these steps address known sources of potential error, they are currently not systematically used because of their dependence on used hardware and reconstruction or proprietary information, limited evidence on their impact, or computational expense.

## Integrated software solutions: from pipelines to neuroinformatics platforms

Building upon decades of method development, many algorithms for the correction of imaging artifacts are now adopted within community-facing software libraries, e.g. DiPY (Garyfallidis et al. 2014), FSL(Jenkinson et al. 2012), MRtrix (J.-D. Tournier et al. 2019), DSIstudio (Yeh 2025), ExploreDTI (Leemans et al. 2009), ANTs (Avants et al. 2011), and others that promote their reliable use. Extensive protocols (Tahedl et al. 2025), user guides, and message boards help users navigate the complex landscape of preprocessing tools and maximize their utility. Layered on top of these libraries, a multitude of preprocessing pipelines have been developed and distributed to lower the barrier to use, mitigate the need for export knowledge to run tools reliably, and promote reproducibility and interoperability. While the list keeps growing, notable examples include QSIprep (Cieslak et al. 2021), Tractoflow (Theaud et al. 2020), PreQual (Cai et al. 2021; Lauzon et al. 2013), idIO (Hsu et al. 2023), Designer (Chen et al. 2024), Tortoise (Irfanoglu et al. 2025), and pyDesigner (Dhiman et al. 2024).

Each of these pipelines implements current state-of-the art and best practices for dMRI preprocessing. They are all publicly-available, actively maintained, and designed for reproducibility. Moreover, the dissemination of containerized versions has advanced the interoperability of such complex pipelines. By standardizing best practices, these tools make it easier to compare results across studies and ensure that conclusions are not confounded by avoidable artifact issues.

Given that the accessibility and utility of complex workflows has been promoted through open science practices and the value of such workflows and their individual steps have been demonstrated by various teams using dedicated experiments, we believe that the era of analyzing uncorrected diffusion data should be considered over. Clinical uptake has historically been slower as advanced preprocessing workflows are not yet routinely integrated into scanner consoles or clinical infrastructures. Encouragingly, more state-of-the art reconstruction and preprocessing are beginning to enter clinical environments through vendor-supported platforms and open interfaces that enable third-party solutions, as well as commercially available end-to-end products, some with regulatory clearance for clinical use.

However, in light of their increased use and distinct differences in the implementation and design of pipelines, additional efforts to benchmark software tools and pipelines to maximize their performance and compatibility across cohorts, protocols, and scanners are still needed. For instance, recent efforts for implicit harmonisation of tools/pipelines identify optimal steps as those ensuring generalisability across scanners/vendors and showing immunity to between-scanner effects (Warrington et al. 2023).

The increasing complexity of the workflows might further impact the computational cost, thereby impacting their accessibility. Certain tools, or their deep learning-based alternatives, provide support for multithreading and/or GPUs to accelerate the calculations, but their implementation requires specialized computing hardware. To maximize accessibility of advanced tools to the global neuroimaging community, various cloud-based neuroinformatics platforms have been developed to integrate data, software, and high-performance computing resources in a single environment (Hayashi et al. 2024; Sherif et al. 2014).

## Maximizing the efficacy of preprocessing by tailoring the data acquisition, reconstruction, and handling

The development of high performance preprocessing tools introduces various prerequisites regarding the data, imaging protocols, and image reconstruction steps. Here we will list some recommendations for data acquisition that would advance the efficacy of their preprocessing. Note that our recommendations are limited to prerequisites for image preprocessing; optimization of tractography itself - including acquisition design and downstream tracking/modeling choices - is beyond the scope of this communication.

*Diffusion-weighting gradient sampled on a sphere*: Under the assumption of polar symmetry of the orientation distribution functions, the diffusion-weighted signal is invariant to the polarity of the diffusion-weighting gradient. Eddy current-induced geometric distortions, their direction in particular, do depend on the polarity. Recently developed tools such as *Eddy* leverage this symmetry and require the acquisition of DW data that has been acquired on the entire sphere instead of a half sphere, an approach that has been commonly adopted in many imaging protocols. While alternative approaches, e.g. ShoreLine *(*Cieslak et al. 2024; Cieslak et al. 2021), don’t have this dependency, we recommend acquiring DW data sampled on full rather than half shells.

*Reverse phase-encoded data*: Most publicly available pipelines rely on the availability of minimally one non-diffusion-weighted image that has been acquired with reversed phase-encoding to correct the susceptibility-induced geometric distortions. If not available, the correction can be performed using (a) a B0 inhomogeneity field map, derived from a dual-echo GRE or (b) a distortion-free structural MRI image. Regardless of the chosen approach, susceptibility-induced geometric distortions cannot be corrected with the dMRI data only and additional data must be acquired.

*Distribution of non-diffusion-weighted images*: Overall it is recommended to acquire dMRI data using optimized gradient encoding schemes. Within this context, it is recommended to intersperse the non-diffusion-weighted or *b*_0_ images uniformly throughout the acquisition. Interspersed *b*_0_ images can increase efficacy of EPI distortion correction of motion-by-susceptibility artifacts (Andersson et al., 2018), are favorable for motion correction and the identification of potential *signal drifts (*Vos et al. 2017).

*Reconstruction*: Where possible, reconstructing magnitude MRI data using techniques that combines all coil data into a single complex image prior to the magnitude operation is preferred over Sum-of-Squares over multiple coil images. Indeed, the latter would amplify the negative impact of *Rician* signal biases on angular contrast and sharpness of the orientation distribution function (Jones and Basser 2004; Sotiropoulos et al. 2013). Ongoing efforts to use real-valued or complex MRI data hold promise to eventually mitigate the impact of Rician signal biases entirely (Eichner et al. 2015).

*Data interpolation*: While we recommend acquiring data with isotropic voxels (Neher et al. 2013; Oouchi et al. 2007), resampling dMRI data to an isotropic or high resolution grid prior to tractography has been shown to improve the delineation of fine white matter pathways, reduce partial volume effects, and enhance the anatomical plausibility of reconstructed fiber tracts (Rheault et al. 2020; Dyrby et al. 2014). While this resampling can be performed during image reconstruction, we recommend reconstructing and preprocessing data at its native resolution, with data resamping being the final step. If not, data resampling data would impact the noise characteristics and the periodicity of Gibbs ringing, thereby lowering the efficiency of dedicated correction strategies.

*BIDS standard*: The adoption of data standards such as the Brain Imaging Data Structure (BIDS) (Gorgolewski et al. 2016) advances the reliable and streamlined use of preprocessing pipelines. The BIDS standard synthesizes critical information regarding data acquisition settings that is required to verify data compatibility with any of the preprocessing steps in a standardized data *json* file. Many pipelines have now adopted the vendor-agnostic BIDS standard to promote the intuitive and streamlined integration of sequence parameters into processing workflows. As more preprocessing methods depend on detailed acquisition metadata (e.g., slice timing/order for slice-to-volume motion correction), continued efforts to ensure converters reliably extract and standardize these fields across vendors and sequences will further improve pipeline robustness.

## Conclusion

The advancement of robust diffusion MRI preprocessing pipelines has elevated the quality of tractography achievable today. Denoising, Gibbs ringing removal, distortion correction, motion/eddy correction, and multi-fiber modeling each contribute vital improvements – increasing SNR, reducing biases, and aligning diffusion data with anatomy. Numerous studies provide evidence that applying these steps yields more reliable, anatomically accurate, and reproducible tractography results than working with unprocessed or minimally processed data. These benefits manifest as higher test-retest consistency, better cross-site harmonization, and more valid reconstructions of known fiber pathways. The primary objective of this paper was to provide an overview of preprocessing methods, but not to provide a comprehensive overview of the various approaches that might be available for any of the artifact corrections. Today, the community now has access to robust, user-friendly pipelines implementing all these best practices. An increasing number of preprocessing pipelines and libraries encapsulate the state-of-the-art methods and are readily available to researchers for free. We urge neuroimaging researchers to take full advantage of these resources and, where possible, revise the image acquisition protocols accordingly. Even as tractography validation is an ongoing endeavor, we should not ignore the substantial progress that has been made in preprocessing. In summary, *did you know that enhancing your diffusion MRI data with these pipelines can improve your tractography?* The literature leaves little doubt. By adopting robust, accessible, and reproducible preprocessing pipelines, we can collectively raise the standard of diffusion MRI research and confidently trust that our maps of brain connectivity are built on the best foundation possible.

## Data Availability

No datasets were generated or analysed during the current study.
